# Antibiotic therapy is associated with an increased incidence of cancer

**DOI:** 10.1007/s00432-022-03998-z

**Published:** 2022-04-19

**Authors:** Christoph Roderburg, Sven H. Loosen, Markus S. Joerdens, Münevver Demir, Tom Luedde, Karel Kostev

**Affiliations:** 1 Clinic for Gastroenterology, Hepatology and Infectious Diseases, Medical Faculty of Heinrich Heine University Düsseldorf, University Hospital Düsseldorf, 40225 Düsseldorf, Germany; 2 Department of Hepatology and Gastroenterology, Charité University Medicine Berlin, Augustenburger Platz 1, 13353 Berlin, Germany; 3 Epidemiology, IQVIA, Frankfurt, Germany

**Keywords:** Antibiotics, Cancer, Malignancy

## Abstract

**Purpose:**

There is a growing body of evidence suggesting the decisive involvement of the human microbiome in cancer development. The consumption of antibiotics may fundamentally change the microbiome and thereby create a precancerous environment promoting cancer development and growth. However, clinical data on the association between the consumption of antibiotics and cancer incidence have remained inconclusive. In this study, we quantified the association between the intake of different antibiotics and various cancer entities among outpatients from Germany.

**Methods:**

This retrospective case–control study based on the IQVIA Disease Analyzer database included 111,828 cancer patients and 111,828 non-cancer controls who were matched to cancer cases using propensity scores. Patients were categorized as non-users, low-consumption (up to 50th percentile), and high-consumption (above 50^th^ percentile) users of antibiotics overall and for each antibiotic class. Multivariable logistic conditional regression models were used to study the association between antibiotic intake within 5 years prior to the index date (first cancer diagnosis for cases or randomly selected date for controls) and cancer incidence.

**Results:**

The probability of cancer was significantly higher among patients with a history of antibiotic intake than in matched controls. Patients using penicillin or cephalosporins displayed a higher incidence of cancer, while the intake of tetracyclines and macrolides actually reduced the risk of cancer development slightly. A complex picture was observed in our cancer site-stratified analyses. Most notably, the consumption of penicillin was significantly and positively associated with cancer development in the respiratory organs only (low consumption OR: 1.33, 95% CI 1.20–1.47; high consumption OR 1.42, 95% CI 1.22–1.64) and cephalosporin consumption was significantly associated with respiratory organ cancer (low consumption OR: 1.32, 95% CI 1.17–1.48, high consumption OR: 1.47, 95% CI 1.29–1.66), breast cancer (high consumption OR: 1.40, 95% CI 1.25–1.56), and lymphoid and hematopoietic tissue cancer (high consumption OR: 1.50, 95% CI 1.35–1.66).

**Conclusion:**

Our data strongly support the hypothesis that the intake of antibiotics is positively associated with the risk of cancer development.

## Introduction

Cancer is a major health threat with about 10.0 million cancer-related deaths in 2020 (Sung et al. 2021). Lung cancer remains the leading cause of cancer-related death, with an estimated 1.8 million deaths (18%), followed by colorectal (9.4%), liver (8.3%), stomach (7.7%), and female breast (6.9%) cancers (Sung et al. 2021). The global cancer burden is expected to be 28.4 million cases in 2040, a 47% rise from 2020, highlighting the critical importance of global cancer control measures aimed at the prevention of the disease (Sung et al. 2021).

Many different risk factors promoting cancer development have been identified, some of which are entity-specific, while others are ubiquitous promoters of cancer (Abbafati et al. 2020). There is increasing evidence indicating that the microbiome may also decisively influence cancer development and progression, as well as the response to therapy (Yu et al. 2021; Rotz and Dandoy 2020). The human microbiome is essential for the correct functioning of many host physiological processes, including metabolic regulation and immune responses (Rook et al. 2017; Cai et al. 2022). Although most studies have focused on the effect of the gut microbiome, many other organs such as the skin, vagina, and lungs harbor their own microbiomes that differ from the gut (González-Sánchez and DeNicola 2021). Tumor development has been associated with dysbiosis not only in the gut but also in the tissue from which the tumor originated. Furthermore, the intratumoral microbiota has a distinct signature in each tumor type (González-Sánchez and DeNicola 2021). It has been hypothesized that antibiotics may play a role in oncogenesis as a result of their effects on the gut microbiome (Ibragimova et al. 2021). Although antibiotics are one of the most commonly prescribed types of medication worldwide (Bitterman et al. 2016; Farooqui et al. 2019), the association between specific antibiotics and various cancer entities has not been clearly delineated. The aim of this study was to fill this gap by providing epidemiologic evidence of an association between antibiotic intake and the risk of cancer development. We used data from the Disease Analyzer database (IQVIA) to examine the potential association between antibiotics and the most common cancers. Our findings may help to adapt prescription strategies to individual patient risk profiles, potentially reducing the incidence of cancer in the future.

## Methods

### Database

This study was based on data from the Disease Analyzer database (IQVIA), which contains drug prescriptions, diagnoses, and basic medical and demographic data obtained directly and in anonymous format from computer systems used in the offices of general practitioners and specialists (Rathmann et al. 2018). The database covers approximately 3% of all outpatient practices in Germany. Diagnoses (according to International Classification of Diseases, 10th revision [ICD-10]), prescriptions (according to Anatomical Therapeutic Chemical [ATC] classification system), and the quality of the reported data is monitored regularly by IQVIA. It has previously been shown that the panel of practices included in the Disease Analyzer database is representative of general and specialized practices in Germany (Rathmann et al. 2018). For example, Rathmann et al. demonstrated good agreement between the incidence or prevalence of cancer diagnoses between the outpatient DA database and German reference data (Rathmann et al. 2018). Finally, this database has already been used in previous studies focusing on cancer (Huber et al. 2020; Jacob et al. 2021).

### Study population

**Fig. 1:**
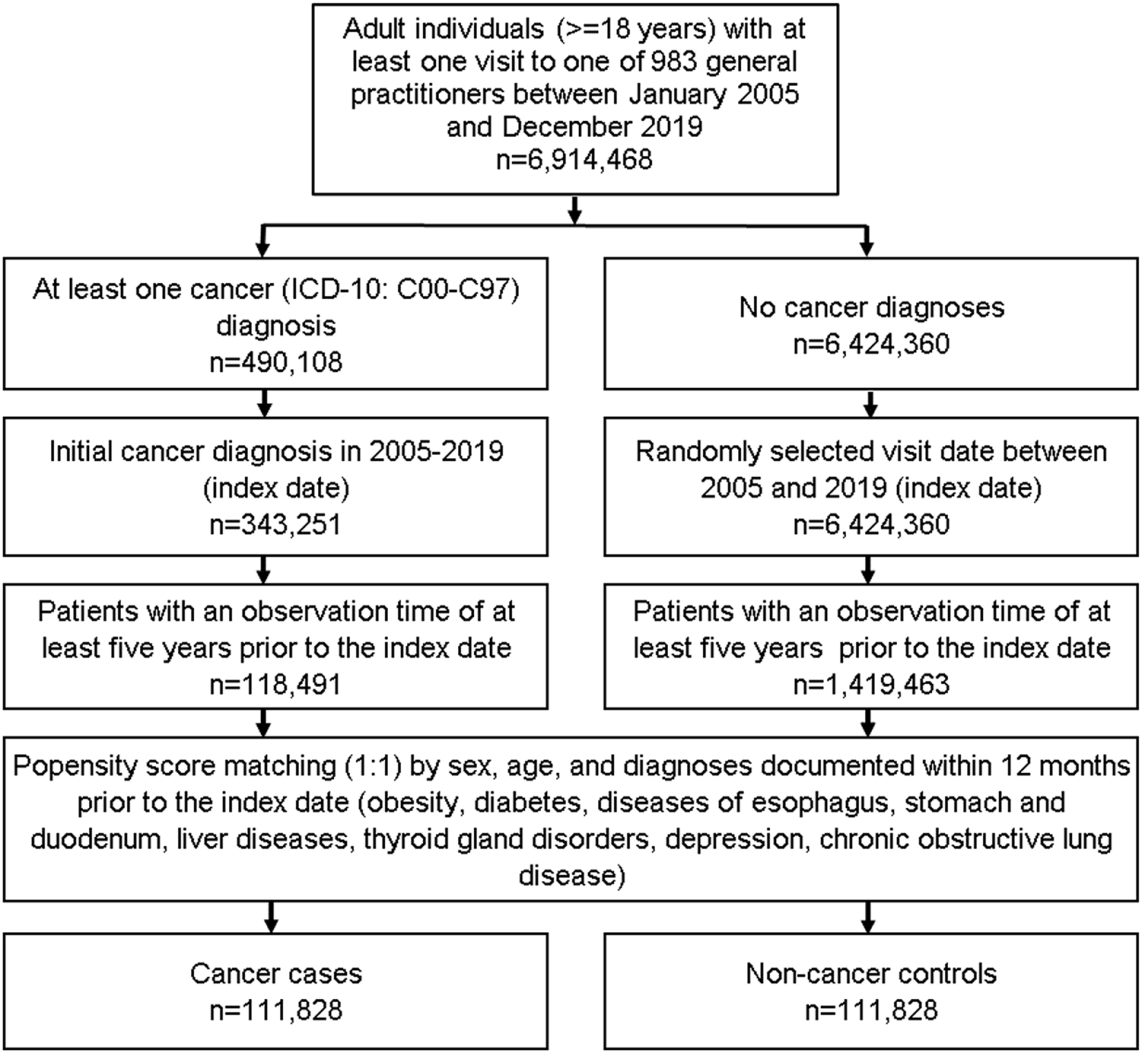
Selection of study patients

This retrospective case–control study included adult patients (≥ 18 years) with an initial cancer diagnosis (ICD-10: C00–C97) in 1274 general practices in Germany between January 2005 and December 2019 (index date; Fig. 1). A further inclusion criterion was an observation time of at least 5 years prior to the index date. Cancer cases were matched to non-cancer controls using greedy nearest neighbor propensity scores based on sex, age, and pre-defined chronic co-diagnoses documented within 5 years prior to the index date (obesity, diabetes, diseases of esophagus, stomach and duodenum, liver diseases, thyroid gland disorders, depression, chronic obstructive lung disease). For the controls, the index date was that of a randomly selected visit between January 2005 and December 2019 (Fig. 1).

### Study outcomes

The main outcome of the study was the association between prescriptions of antibiotic drugs and the incidence of cancer diagnosis. The antibiotics included were tetracyclines (ATC codes J01A), penicillins (ATC codes J01C), cephalosporins (ATC codes J01D), sulfonamides and trimethoprim (ATC codes J01E), macrolides (ATC codes J01F), and quinolones (ATC codes J01M). The total count of milligrams (mg) prescribed within 5 years prior to the index date was calculated for each patient. Patients were categorized as non-users, low-consumption (up to 50th percentile), and high-consumption (above 50th percentile) users of antibiotics overall and for each antibiotic class.

### Statistical analyses

Differences in the sample characteristics between those with and those without cancer were assessed using chi-squared tests for categorical variables and Wilcoxon tests for age. A multivariable conventional logistic regression model was applied to study the association between antibiotic consumption (yes/no), antibiotic amount (low-consumption users and high-consumption users vs. non-users) and cancer. The models were multivariable models as they included all antibiotic drug classes because many individuals received several drug classes during the follow-up time. In such cases, the effect of each drug class was adjusted for the other drug classes. Since adjustment for the same variables which were used as matching factors did not change the effects of antibiotic classes, we did not use matching factors for the adjustment.

Regression models were calculated for all patients and were also stratified by sex and five age groups (age 18–50, age 51–60, age 61–70, age 71–80, age > 80). Finally, these models were applied for each of the most common cancer sites including digestive organs (ICD-10: C15–C26), respiratory organs (ICD-10: C30–C39), breast (ICD-10: C50), prostate (ICD-10: C61), skin (ICD-10: C43, C44), urinary tract (ICD-10: C64–C68), and lymphoid and hematopoietic tissue (ICD-10: C81–C96) versus matched non-cancer patients. Due to the large number of different comparisons and large patient numbers, a *p* value of < 0.001 was considered statistically significant. Analyses were carried out using SAS version 9.4 (SAS Institute, Cary, USA).

## Results

### Basic characteristics of the study sample

The present study included 111,828 patients with cancer and 111,828 individuals without cancer. The basic characteristics of study patients are displayed in Table 1. The mean age [SD] was 67.6 (Bach et al. 2020) years and 50.8% of subjects were women. No significant differences were observed between the cancer and the control group in terms of the frequency of co-diagnoses, with diseases of the esophagus, stomach/ duodenum, thyroid gland, and type 2 diabetes being the most common disorders in both groups.

**Table 1:** Baseline characteristics of the study sample after 1:1 propensity score matching

Variable	Proportion affected among patients with cancer (%) *N* = 111,828	Proportion affected among patients without cancer (%) *N* = 111,828	*P* value
Age (mean, SD)	67.6 (13.6)	67.6 (13.7)	0.236
Age ≤ 50	11.2	11.6	0.087
Age 51**–**60	17.1	17.1	
Age 61**–**70	24.0	23.9	
Age 71**–**80	30.7	30.3	
Age > 80	17.0	17.1	
Female	50.8	50.8	1.000
Male	49.2	49.2	
Diabetes	20.2	20.2	1.000
Obesity	6.1	6.1	1.000
Diseases of esophagus, stomach, and duodenum	26.0	26.0	1.000
Liver diseases	7.7	7.7	1.000
Thyroid gland disorders	18.6	18.6	1.000
Chronic obstructive lung disease	10.8	10.8	1.000
Depression	14.1	14.1	1.000

Proportions of patients are given in % unless otherwise indicated

*SD* standard deviation

### Association of antibiotic consumption with cancer

We first performed a logistic regression analysis to identify a possible association between the consumption of antibiotics and cancer development at any site. In these analyses, high consumption (OR: 1.13, 95% CI 1.12–1.15) of antibiotics was significantly associated with increased odds of cancer development compared to non-users, while low consumption (OR: 1.02, 95% CI 0.98–1.06) was not. Interestingly, the cancer-promoting effect was observed for cephalosporins (OR: 1.13, 95% CI 1.10–1.17 for low consumption, and OR: 1.23, 95% CI 1.18–1.27 for high consumption)) and quinolones (OR: 1.08, 95% CI 1.05–1.11 for low consumption and OR: 1.16, 95% CI 1.12–1.20 for high use) while high consumption of tetracyclines (OR: 0.92, 95% CI 0.87–0.97) and low consumption of macrolides (OR: 0.95, 95% CI 0.92–0.97) actually reduced the risk of cancer development slightly (Table 2).

**Table 2:** Association between antibiotic therapy and cancer (multivariable logistic regression models)

Antibiotic therapy	Proportion among patients with cancer	Proportion among patients without cancer	OR (95% CI)	*P* value
Any antibiotic drug				
No therapy	55.2	56.0	Reference	
≤ 6000 mg	16.8	16.9	1.02 (0.98–1.06)	0.386
> 6000 mg	28.0	25.1	1.13 (1.12–1.15)	< 0.001
Tetracyclines (J01A)				
No therapy	93.6	93.6	Reference	
≤ 2000 mg	4.2	4.1	0.98 (0.94–1.02)	0.401
> 2000 mg	2.2	2.3	0.92 (0.87–0.97)	0.003
Penicillins (J01C)				
No therapy	85.7	86.7	Reference	
≤ 20,000 mg	10.1	9.5	1.05 (1.02–1.08)	0.002
> 20,000 mg	4.2	3.8	1.05 (1.00–1.09)	0.043
Cephalosporins (J01D)				
No therapy	86.0	88.1	Reference	
≤ 6000 mg	7.9	6.9	1.13 (1.10–1.17)	< 0.001
> 6000 mg	6.1	5.0	1.23 (1.18–1.27)	< 0.001
Sulfonamides and trimethoprim (J01E)				
No therapy	96.5	96.9	Reference	
≤ 9600 mg	2.3	2.1	1.03 (0.98–1.10)	0.257
> 9600 mg	1.2	1.0	1.06 (0.98–1.14)	0.172
Macrolides (J01F)				
No therapy	83.4	84.0	Reference	
≤ 3000 mg	8.7	8.9	0.95 (0.92–0.97)	< 0.001
> 3000 mg	7.9	7.1	1.04 (1.00–1.07)	0.042
Quinolones (J01M)				
No therapy	81.9	84.0	Reference	
≤ 5000 mg	10.5	9.7	1.08 (1.05–1.11)	< 0.001
> 5000 mg	7.6	6.3	1.16 (1.12–1.20)	< 0.001

### Age- and sex-stratified analyses

Next, we attempted to evaluate the potential age- or sex-related associations between the consumption of antibiotics and cancer. Here, the positive association between high antibiotic consumption overall as well as high or low consumption of cephalosporins and high consumption of quinolones was observed in both women and men and across all age groups, with no significant sex- or age-related differences (Table 3). The negative association between high consumption of tetracyclines and cancer was only observed in women and in patients aged between 61 and 70 years, while the negative association between macrolides and cancer was only observed only in patients under 50 and those aged between 61 and 70 (Table 3).

**Table 3:** Association between antibiotic therapy and cancer by sex and age group (multivariable logistic regression models)

Antibiotic therapy	OR (95% CI)						
	Women (*n* = 113,532)	Men (*n* = 110,124)	Age ≤ 50 (*n* = 25,446)	Age 51–60 (*n* = 38,214)	Age 61–70 (*n* = 53,544)	Age 71–80 (*n* = 68,266)	Age > 80 (*n* = 38,186)
Any antibiotic drug							
≤ 6000 mg	1.03 (0.99–1.06)	1.06 (1.02–1.09)*	1.00 (0.94–1.07)	1.01 (0.96–1.06)	1.03 (0.98–1.08)	1.05 (1.01–1.10)	1.11 (1.05–1.18)*
> 6000 mg	1.16 (1.13–1.19)*	1.19 (1.16–1.22)*	1.13 (1.07–1.20)*	1.19 (1.13–1.25)*	1.21 (1.17–1.26)*	1.14 (1.10–1.18)*	1.21 (1.16–1.28)*
Tetracyclines (J01A)							
≤ 2000 mg	0.95 (090–1.01)	1.02 (0.96–1.08)	0.99 (0.89–1.11)	0.97 (0.88–1.07)	0.99 (0.91–1.08)	1.00 (0.92–1.08)	0.95 (0.85–1.06)
> 2000 mg	0.90 (0.83–0.97)*	0.94 (0.87–1.02)	0.93 (0.79–1.10)	0.86 (0.75–0.97)	0.82 (0.73–0.92)*	1.02 (0.92–1.13)	0.99 (0.84–1.17)
Penicillins (J01C)							
≤ 20,000 mg	1.04 (1.00–1.08)	1.05 (1.01–1.10)	1.06 (0.98–1.14)	1.13 (1.05–1.20)*	1.09 (1.03–1.16)*	0.98 (0.93–1.03)	1.02 (0.95–1.09)
> 20,000 mg	1.05 (0.99–1.11)	1.04 (0.98–1.11)	1.04 (0.94–1.16)	1.07 (0.97–1.18)	1.17 1.07–1.28)*	0.97 (0.89–1.06)	1.00 (0.89–1.12)
Cephalosporins (J01D)							
≤ 6000 mg	1.15 (1.10–1.20)*	1.12 (1.07–1.17)*	1.17 (1.07–1.28)*	1.15 (1.07–1.25)*	1.16 (1.08–1.24)*	1.09 (1.02–1.16)*	1.14 (1.06–1.22)*
> 6000 mg	1.29 (1.23–1.36)*	1.16 (1.10–1.23)*	1.38 (1.24–1.52)*	1.23 (1.13–1.34)*	1.20 (1.11–1.30)*	1.20 (1.11–1.29)*	1.22 (1.11–1.33)*
Sulfonamides and trimethoprim (J01E)							
≤ 9600 mg	1.03 (0.97–1.10)	1.13 (1.00–1.28)	0.93 (0.79–1.11)	1.08 (0.92–1.26)	1.05 (0.92–1.20)	1.07 (0.96–1.18)	1.07 (0.95–1.20)
> 9600 mg	1.04 (0.95–1.15)	1.18 (1.01–1.37)*	1.16 (0.89–1.50)	1.10 (0.90–1.35)	0.99 (0.82–1.19)	1.10 (0.96–1.27)	1.06 (0.90–1.24)
Macrolides (J01F)							
≤ 3000 mg	0.96 (0.92–1.00)	0.94 (0.90–0.98)	0.90 (0.84–0.98)*	0.93 (0.87–1.00)	0.95 (0.89–1.0)	0.95 (0.90–1.01)	1.02 (0.94–1.11)
> 3000 mg	1.03 (0.99–1.08)	1.04 (0.99–1.09)	0.96 (0.89–1.05)	1.04 (0.97–1.12)	1.10 (1.03–1.18)*	0.99 (0.93–1.05)	1.12 (1.02–1.22)
Quinolones (J01M)							
≤ 5000 mg	1.03 (0.99–1.07)	1.16 (1.11–1.21)*	0.97 (0.89–1.06)	1.07 (1.00–1.15)	1.06 (1.00–1.13)	1.11 (1.05–1.16)*	1.15 (1.08–1.23)*
> 5000 mg	1.14 (1.09–1.20)*	1.19 (1.13–1.26*	1.12 (1.00–1.24)	1.17 (1.07–1.27)*	1.16 (1.08–1.24)*	1.19 (1.12–1.27)*	1.16 (1.07–1.25)*

*p < 0.001

*OR* odds ratio, *CI* confidence interval

### Associations with defined cancer sites

Finally, we aimed at further evaluating the potential association between different antibiotics and the different cancer sites. Notably, the consumption of penicillin was significantly and positively associated with cancer of the respiratory organs only (low consumption OR: 1.33, 95% CI: 1.20–1.47; high consumption OR 1.42, 95% CI 1.22–1.64, Table 4). Low penicillin consumption was also associated with slightly increased odds of lymphoid and hematopoietic tissue cancers (OR: 1.17, 95% 1.09–1.27, Table 4). Cephalosporin consumption was significantly associated with respiratory organ cancer (low consumption OR: 1.32, 95% CI 1.17–1.48, high consumption OR: 1.47, 95% CI 1.29–1.66), breast cancer (high consumption OR: 1.40, 95% CI 1.25–1.56), and lymphoid and hematopoietic tissue cancer (high consumption OR: 1.50, 95% CI 1.35–1.66). Sulfonamides and trimethoprim were significantly associated with urinary tract cancer (low use OR: 1.79, 95% CI 1.42–2.25; high consumption OR 1.74, 95% CI 1.28–2.36) and lymphoid and hematopoietic tissue cancer (high consumption OR: 1.31, 95% CI 1.07–1.60, Table 4). Finally, quinolone consumption was associated with higher odds of prostate cancer (low consumption OR: 1.21, 95% CI 1.10–1.33; high consumption OR 1.47, 95% CI 1.31–1.66), urinary tract cancer (low consumption OR: 1.64, 95% CI 1.38–1.71; high consumption OR 1.57, 95% CI 1.38–1.78), and lymphoid and hematopoietic tissue cancer (high use OR: 1.20, 95% CI 1.09–1.32, Table 4). By contrast, the consumption of high-dose tetracyclines was negatively associated with the incidence of urinary tract cancer (OR: 0.70, 95% CI 0.56–0.88, Table 4). In summary, these data further underscore the fact that the association between cancer development and the consumption of antibiotics is complex and influenced by many different co-factors.

**Table 4:** Association between antibiotic therapy and cancer by cancer sites (multivariable logistic regression models)

Antibiotic therapy	OR (95% CI)						
	Digestive organs (*n* = 33,174)	Respiratory organs (*n* = 15,392)	Prostate (*n* = 22,496)	Breast (*n* = 29,354)	Skin (*n* = 45,680)	Urinary tract (*n* = 14,644)	Lymphoid and hematopoietic tissue (*n* = 28,184)
Any antibiotic drug							
≤ 6000 mg	1.03 (0.97–1.09)	1.16 (1.06–1.27)*	1.10 (1.03–1.20)*	0.97 (0.91–1.03)	1.02 (0.97–1.07)	1.14 (1.04–1.25)*	1.02 (0.96–1.09)
> 6000 mg	1.09 (1.03–1.14)*	1.44 (1,34–1.55)*	1.17 (1.10–1.25)*	1.08 (1.02–1.14)	1.09 (1.04–1.13)	1.32 (1.22–1.42)*	1.35 (1.28–1.42)*
Tetracyclines							
≤ 2000 mg	0.93 (0.83–1.04)	1.07 (0.92–1.24)	0.99 (0.86–1.14)	0.92 (0.82–1.04)	1.00 (0.91–1.09)	1.02 (0.85–1.21)	1.04 (0.92–1.16)
> 2000 mg	0.95 (0.81–1.11)	0.78 (0.63–0.95)	0.96 (0.81–1.15)	0.93 (0.80–1.11)	0.97 (0.86–1.09)	0.70 (0.56–0.88)*	1.02 (0.87–1.20)
Penicillins							
≤ 20,000 mg	1.02 (0.94–1.10)	1.33 (1.20–1.47)*	0.92 (0.84–1.02)	0.99 (0.91–1.07)	0.98 (0.92–1.05)	0.96 (0.86–1.08)	1.17 (1.09–1.27)*
> 20,000 mg	0.98 (0.88–1.11)	1.42 (1.22–1.64)*	0.92 (0.79–1.07)	0.89 (0.79–1.02)	1.10 (1.00–1.21)	0.91 (0.77–1.08)	1.10 (0.97–1.23)
Cephalosporin**s**							
≤ 6000 mg	1.06 (0.98–1.16)	1.32 (1.17–1.48)*	1.03 (0.92–1.15)	1.13 (1.03–1.24)	1.17 (1.09–1.26)	1.21 (1.06–1.37)	1.13 (1.03–1.23)
> 6000 mg	1.14 (1.03–1.26)	1.47 (1.29–1.66)*	1.01 (0.89–1.15)	1.40 (1.25–1.56)*	1.11 (1.03–1.21)	1.14 (0.98–1.32)	1.50 (1.35–1.66)*
Sulfonamides and trimethoprim							
≤ 9600 mg	0.89 (0.76–1.04)	1.01 (0.80–1.27)	1.18 (0.91–1.53)	1.01 (0.87–1.16)	1.13 (0.99–1.28)	1.79 (1.42–2.25)*	0.87 (0.74–1.02)
> 9600 mg	1.06 (0.85–1.32)	0.74 (0.53–1.01)	1.13 (0.78–1.63)	1.16 (0.95–1.42)	0.85 (0.72–1.01)	1.74 (1.28–2.36)*	1.31 (1.07–1.60)*
Macrolides							
≤ 3000 mg	0.97 (0.90–1.05)	1.01 (0.91–1.13)	1.00 (0.90–1.10)	0.91 (0.84–0.99)	0.99 (0.93–1.05)	0.91 (0.80–1.03)	0.98 (0.90–1.06)
> 3000 mg	1.00 (0.92–1.09)	1.15 (1.02–1.28)	1.09 (0.96–1.22)	1.07 (0.98–1.17)	0.98 (0.91–1.05)	0.90 (0.78–1.03)	1.20 (1.10–1.31)*
Quinolones							
≤ 5000 mg	1.12 (1.04–1.20)	1.11 (1.00–1.24)	1.21 (1.10–1.33)*	0.92 (0.85–1.00)	1.00 (0.94–1.07)	1.64 (1.38–1.71)*	1.01 (0.93–1.09)
> 5000 mg	1.16 (1.06–1.27)	1.07 (0.95–1.20)	1.47 (1.31–1.66)*	1.09 (0.99–1.20)	0.99 (0.91–1.06)	1.57 (1.38–1.78)*	1.20 (1.09–1.32)*

*p < 0.001

*OR* odds ratio, *CI* confidence interval

## Discussion

In recent years, it has become increasingly obvious that the consumption of antibiotics may play an important role in the development of cancer. Both experimental and clinical analyses have demonstrated an increased incidence of cancer in patients with a history of antibiotic intake, but results have remained inconclusive (Bach et al. 2020; Bagley et al. 2017; Ramírez-Labrada et al. 2020; Wertman et al. 2021; Bi et al. 2021; Cheung et al. 2021; Amadei and Notario 2020; Martins Lopes et al. 2020). Using the population-based Disease Analyzer database (IQVIA) (Rathmann et al. 2018), we demonstrate that the probability of cancer is significantly higher among patients with a history of antibiotic intake. This effect was especially striking in patients receiving penicillins and cephalosporins, while tetracyclines and macrolides appeared to protect against the development of cancer. Our analyses stratified by cancer site revealed an even more complex picture. Here, the consumption of penicillins was significantly and positively associated with cancer of the respiratory organs and, in the case of low consumption, also with cancers of the lymphoid and hematopoietic tissue, while the intake of cephalosporin was significantly associated with respiratory organ cancer, breast cancer, and cancer of the lymphoid and hematopoietic tissue, highlighting a potentially distinctive pathophysiological connection between different classes of antibiotics and different cancer entities.

Cancer is a multifactorial disease with a complex pathophysiology. Genetic causes have been studied extensively in past; however, it has recently become more obvious that many cancer cases are even more closely associated with environmental factors or are associated equally with a combination of both (Wu et al. 2018). For example, human papilloma virus infection and tobacco smoking are responsible for up to 90% of cervical squamous cell carcinomas and lung cancers, respectively (Lewandowska et al. 2019). Recent epidemiological studies have established the commensal microbiota as a previously neglected modulator of carcinogenesis, immune response, and response to anti-cancer therapy (Bhatt et al. 2020; Fessler et al. 2019; Raza et al. 2019). Today, various authors theorize that individual microbial pathogens contribute to cancer development in approximately 15–20% of all cases (González-Sánchez and DeNicola 2021; Martel et al. 2012). It has been suggested that different lifestyle factors that have been found to be associated with an elevated risk of cancer development might act via microbiota-related mechanisms (González-Sánchez and DeNicola 2021). In this context, it was hypothesized that the intake of antibiotics, which are related to profound and long-term changes in the human microbiome, might be associated with an increased risk of various forms of neoplasia. Supporting this hypothesis, our findings show that the probability of cancer in general is significantly higher among patients with a history of antibiotic intake than in matched controls. In this context, recent studies have suggested that cancer initiation and progression are complex processes that are impacted in a very specific manner by global changes in the microbiome rather than by single pathogens (González-Sánchez and DeNicola 2021; Bhatt et al. 2017; Schwabe and Jobin 2013). In line with this, we demonstrate that while penicillins and cephalosporins increase the odds of cancer, tetracyclines and macrolides may instead have a preventive effect with respect to cancer. Notably, these data are in line with recent data on colorectal cancer (CRC) showing that the intake of both penicillins and cephalosporins is associated with colorectal cancer while the consumption of tetracyclines is not (Simin et al. 2020). By contrast, no such differences between different antibiotics were found for breast cancer, in which tetracycline actually had the strongest cancer-promoting effect, underscoring the complexity of the association between antibiotics and cancer.

The vast majority of human microbiota reside in the gastrointestinal tract, particularly in the colon, and can interact both locally and systemically with cancer cells. Consequently, most epidemiological analyses in the past have focused on this interaction. Aneke-Nash and colleagues recently performed a meta-analysis of six studies providing 16 estimates of the association between the level of antibiotic consumption and colorectal neoplasia and showing that individuals with the highest levels of antibiotic exposure had a 10% higher risk of colorectal neoplasia than those with the lowest exposure (Aneke-Nash et al. 2021). In addition, this effect differed between broad- and narrow-spectrum antibiotics, and possibly within the colorectal continuum. Due to the small sample sizes available, we are unable to draw conclusions about the antibiotic-related cancer risk specifically in the colorectum. Beyond colorectal cancer, we also provide a comprehensive analysis on the association between the intake of antibiotics and many different cancer entities by analyzing > 220,000 patients from a large population-based database in Germany. We also demonstrate an elevated risk of non-digestive tract cancers in patients with a history of antibiotic consumption. Notably, specific microbial populations have been described for many organs, revealing a different microbiome signature for each (summarized in González-Sánchez and DeNicola (2021)). Clearly, then, antibiotic-induced alterations in the local microbiome are likely to play a key role in the development of cancer in organs distant from the gut, which is in line with our findings. Of course, a potential bias must not be forgotten when considering the cause of the antibiotic-associated increase in cancer rates suggested by our data. We cannot exclude the possibility that the accumulation of patients suffering from certain diseases that frequently lead to antibiotic consumption and are associated with cancer development may amplify the effect. Examples of such diseases include ulcerative colitis and primary sclerosing cholangitis.

We acknowledge the fact that our study is subject to various limitations, most of which are due to the chosen study design and cannot be avoided (Labenz et al. 2020a; Loosen et al. 2021; Roderburg et al. 2021). Most importantly, diagnoses within our database are coded as ICD-10 codes, which might be associated with the misclassification of certain diagnoses. In addition, data might be incomplete for certain patients; in particular, information regarding lab values or drug intake was not available for all patients, leading to their exclusion from this analysis (Table 1). Furthermore, data on the socioeconomic status (e.g., education and income of patients) as well as lifestyle-related risk factors (e.g., smoking, alcohol consumption, and physical activity) are lacking within the Disease Analyzer database and thus cannot be taken into account in our study. However, the IQVIA Disease Analyzer database used for the present analyses has been used extensively for various academic publications (e.g., (Huber et al. 2020; Jacob et al. 2021; Labenz et al. 2020b)) and its validity has been well demonstrated (Rathmann et al. 2018). We also want to highlight the fact that a selection bias must be assumed due to the fact that patients who take antibiotics more frequently presumably have more frequent contact with their physicians than those who take no antibiotics. Such a bias might also explain the finding that the step to the minimum observation time of 5 years prior to the index date reduces the number of persons to about 1/3 in cancer cases but 1/5 in non-cancer cases. This may be due to the fact that cancer patients had presumably attended the doctor for a greater number of diseases previously and were, therefore, observed for a longer period or more often and that patients with a longer medical history have a better chance of having cancer detected. Next, subgroup analyses of individual cancer sites (e.g., left/ right sided colorectal cancer) were not feasible due to the small sample sizes available. We, therefore, grouped different tumor entities with similar pathomechanisms (e.g., digestive or respiratory organs), which might be associated with a presentation bias as described in detail in a recent study (Loosen et al. 2021). Finally, the information regarding antibiotic consumption was only available for a sufficient number of patients for a 5-year period, meaning that it was not possible to assess the influence of longer periods of antibiotic consumption, potentially leading to an underestimation of the association.

In summary, by analyzing data from a large German primary care provider database, we demonstrate that the intake of various antibiotics is associated with an increased risk of cancer in a dose- and tumor site-specific manner, irrespective of patients’ age and sex. Thus, along with previous data, our study including > 220,000 patients suggests that the clinical management of patients needing antibiotics should include a careful and structured risk assessment for the development of cancer to improve long-term outcomes in these patients. For example, patients with high/frequent consumption of penicillins or cephalosporins might be presented in a specific “board” and discussed with dedicated infectiologists and oncologists, as was recently suggested in the context of specific antibiotic stewardship programs.
